# Pseudocyst in ectopic pancreas: diagnosis and percutaneous treatment guided by MDCT

**DOI:** 10.1590/0100-3984.2016.0194

**Published:** 2018

**Authors:** Camila Bastos Lapa, Eduardo Cesar Freire, João Maurício Canavezi Indiani, Marcelo Fontalvo Martins, Marcelo Souto Nacif

**Affiliations:** 1 URC Diagnóstico por Imagem, São José dos Campos, SP, Brazil.; 2 Universidade Federal Fluminense (UFF), Niterói, RJ, Brazil.

*Dear Editor,*

**Figure 1:**
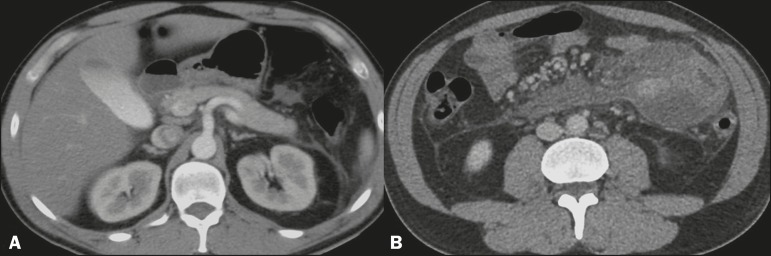
Axial MDCT scan of the entire abdomen, after intravenous administration of an iodinated contrast agent, in the arterial and portal phases (**A** and **B**, respectively). Note the pancreas in its usual location, without signs of inflammation (in **A**). In **B**, note the ectopic pancreatic tissue, located in the mesentery, in close contact with the proximal segment of the jejunal A B loop, with adjacent fluid.

**Figure 2:**
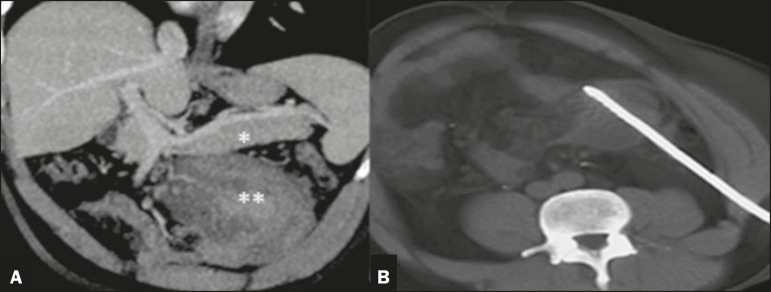
Oblique coronal MDCT scan of the entire abdomen, in the portal phase (**A**), in which the pancreas can be seen in its usual location (asterisk), the ectopic pancreas showing similar density and signs of inflammation (double asterisk). In **B**, puncture and MDCTA B guided percutaneous drainage.

A 40-year-old man presented with a 12-h history of severe abdominal pain, nausea, and vomiting. Although he reported no comorbidities, he stated that he had concomitant constipation and had consumed alcoholic beverages over the past three days. Physical examination revealed pain on palpation of the lower abdomen. Multidetector computed tomography (MDCT) of the abdomen showed a normal pancreas and tissue formation with a density of 30 HU, similar to that of the pancreatic parenchyma (Figure 1), located in the mesentery, in close contact with the proximal segment of the jejunal loop, measuring 2.8 × 2.9 × 2.9 cm, with adjacent liquid (Figures 1 and 2). The patient was hospitalized, with high levels of amylase and lipase, being treated with nutritional support and antibiotic coverage. His pain worsened, persisting for 12 more days. Another MDCT scan showed the formation of a pseudocapsule, with contrast enhancement and residual adjacent fluid. To look for infection, we opted for percutaneous drainage, smear cytology, and determination of the amylase level in the liquid (Figure 2). Cytometry showed the presence of leukocytes, a differential count with a predominance of mononuclear cells (60% lymphocytes), and the absence of malignancy. The Gram stain was negative, as were tests for fungi, acid-fast bacilli, and other bacteria. The pH was 7.79, the LDH level was 405 IU/mL, and the amylase level was 1207 IU/L. The post-drainage evolution was favorable, and the patient was discharged in good clinical condition. At this writing, he has been in outpatient follow-up for six months, during which time he has been asymptomatic.

Tumors and pseudotumors of the upper abdomen have been the subject of recent studies in the radiology literature of Brazil^(^^1^^-^^7^^)^. Ectopic pancreas is a rare condition that is most common in males between the fourth and sixth decades of life. It is defined as pancreatic tissue in an anomalous location, with no anatomical, neural, or vascular connection with the normal pancreas^(^^8^^)^. Although the pathogenesis of ectopic pancreas is unknown, there are two hypotheses: the first suggests that there is transplantation of embryonic pancreatic cells to neighboring structures during the intestinal rotation process; and the second proposes that embryonic buds remain attached to the primitive duodenum and, during the growth and formation of the gastrointestinal tract, could be carried to sites proximal or distal to the primitive duodenum^(^^9^^)^.

The majority of patients with ectopic pancreas are asymptomatic, and the diagnosis is generally made on the basis of an incidental finding, either during an imaging examination or during exploratory surgery motivated by other diseases^(^^10^^,^^11^^)^. It is important to note that ectopic pancreatic tissue is susceptible to all of the same diseases that effect the native pancreas^(^^12^^)^.

The treatment of ectopic pancreas is directly related to the symptoms and degree of malignancy. Resection is recommended for symptomatic patients with lesions greater than 3.0 cm and possible malignancy. However, when the lesion is smaller than 3.0 cm or is an incidentaloma, there is still no consensus regarding the choice between resection and conservative percutaneous treatment with periodic surveillance. Resection of an ectopic pancreas can be performed endoscopically or surgically^(^^13^^)^.

In conclusion, we believe that, although rare, a pseudocyst^(^^14^^)^ in ectopic pancreatic tissue should be addressed in order to avoid neoplasia and infection, facilitating clinical treatment and periodic surveillance of the patient, in which the radiologist plays an essential role.
